# Editorial: Systems Biological Aspects of Pituitary Tumors

**DOI:** 10.3389/fendo.2016.00086

**Published:** 2016-06-30

**Authors:** Xianquan Zhan, Dominic M. Desiderio

**Affiliations:** ^1^Key Laboratory of Cancer Proteomics of Chinese Ministry of Health, Xiangya Hospital, Central South University, Changsha, China; ^2^Hunan Engineering Laboratory for Structural Biology and Drug Design, Xiangya Hospital, Central South University, Changsha, China; ^3^State Local Joint Engineering Laboratory for Anticancer Drugs, Xiangya Hospital, Central South University, Changsha, China; ^4^The State Key Laboratory of Medical Genetics, Central South University, Changsha, China; ^5^The Charles B. Stout Neuroscience Mass Spectrometry Laboratory, Department of Neurology, College of Medicine, University of Tennessee Health Science Center, Memphis, Tennessee, USA

**Keywords:** pituitary adenoma, systems biology, omics, molecular networks, chemokine network, calcium–phosphate homeostasis

**The Editorial on the Research Topic**

**Systems Biological Aspects of Pituitary Tumors**

Pituitary adenomas are a category of neoplasms with a high degree of heterogeneity that occur in the central regulatory organ pituitary, which plays important roles in the hypothalamus–pituitary-targeted organ axis systems that impact on important physiological functions of human body (1–3). Rapidly developed omics and systems biology (4–6) impact on treatment of pituitary adenomas and gradually change the paradigms from the traditional single-factor strategy to a multi-parameter systematic strategy. A pituitary adenoma is a complex, chronic, and whole-body disease that alters the genome, transcriptome, proteome, and metabolome and involves multiple factors, processes, and consequences (7–9). Pituitary adenomas gradually change in the model of predictive screening, diagnosis, and prognostic assessment of pituitary adenomas that previously only depended on changes of serum single-hormone change and pituitary imaging, and in the therapeutic model of cancer from general radiotherapy and chemotherapy to a personalized strategy (8, 9).

This present issue focuses on systems biological aspects of pituitary adenomas, which contains four topics. (i) The first topic addressed vitamin D status and calcium–phosphate homeostasis in acromegaly patients (Halupczok-Żyła et al.). Vitamin D deficiency and alteration in calcium–phosphate balance are associated with a wide spectrum of diseases, such as cancer, diabetes, cardiovascular disease, and respiratory disease. Data demonstrate that acromegaly patients are at the higher risk of vitamin D deficiency and have a tendency to a lower level of calcium and higher level of inorganic phosphate. Data suggest the importance of inorganic calcium–phosphate homeostasis in the pathogenesis of acromegaly patients (growth hormone pituitary adenomas) from the systemic view point. (ii) The second topic addressed the alteration in the chemokine network in pituitary adenomas from a systemic view (Grizzi et al.). Chemokines are a category of inflammatory mediators that exert their roles through typical and atypical chemokine receptor signaling pathways. An alteration of chemokines and receptors is associated with cancer and inflammatory diseases. The chemokine network is proposed as the target of biomarker and new therapeutic approach for pituitary adenomas. (iii) The third topic addressed the proteomic variations in pituitary adenomas (Zhan and Wang). Proteomics is the key component of functional genomics and systems biology. Much progress has been achieved in pituitary adenoma proteomics to expand and enrich the systems biology analysis of pituitary adenomas. This topic emphasized the significance of variations in proteome and protein molecular networks for personalized and precise studies of pituitary adenomas. (iv) The fourth topic focused on molecular network variations in different subtypes of non-functional pituitary adenomas (Zhan and Long). That topic emphasized the integrative molecular networks derived from multiple omics data at the genome, transcriptome, proteome, peptidome, and metabolome levels, and on their variations in different subtypes of non-functional pituitary adenomas. These studies will benefit discovery of effective and reliable biomarkers and therapeutic targets for personalized and precise studies of highly heterogeneous non-functional pituitary adenomas.

It is clear that the systems biological aspects of pituitary adenomas cover a very wide range from systemic concepts to analytic methods; from genome, transcriptome, proteome, and metabolome to interactome; from single omics to integrative omics; from panel analysis to molecular network analysis; from genetic feature to phenotype; and from common features to individual characteristics, to investigate the diversity of pituitary adenomas that include functional and non-functional pituitary adenomas and their subtypes (10–15). It must be clearly mentioned that this issue contains only a limited fraction of the very important systems biological aspects of pituitary adenomas.

This research topic serves as a modest spur to induce researchers who study systems biology strategies to come forward with its valuable contributions to research and clinical practice of pituitary adenomas.

From the point of view of systematic strategies in pituitary adenomas, it is necessary for future studies to systematically investigate variations in the genome, transcriptome, proteome, peptidome, and metabolome in pituitary adenoma tissue and body-fluids (cerebrospinal fluid, CSF; serum/plasma), and especially for different subtypes of pituitary adenomas (7, 9). Systems biology approaches will be used to integrate all experimental data and all clinical information of an individual and to propose corresponding molecular networks specific to a pituitary adenoma in order to achieve efficient prediction screening, early stage diagnosis, prognostic assessment, and individualized and precise prevention and therapy (16, 17).

Future issues will collect different levels of omics studies, especially the integrative omics studies together with genetics and clinical information with developed advanced computational biology approaches.

## Author Contributions

All authors listed have made substantial, direct, and intellectual contribution to the work and approved it for publication.

## Conflict of Interest Statement

The authors declare that the research was conducted in the absence of any commercial or financial relationships that could be construed as a potential conflict of interest.
